# School Bus Rebate Program and Student Educational Performance Test Scores

**DOI:** 10.1001/jamanetworkopen.2024.3121

**Published:** 2024-03-20

**Authors:** Meredith Pedde, Adam Szpiro, Richard A. Hirth, Sara D. Adar

**Affiliations:** 1 Department of Epidemiology, University of Michigan, Ann Arbor, Michigan; 2 Department of Biostatistics, University of Washington, Seattle, Washington; 3 Department of Health Management and Policy, University of Michigan, Ann Arbor, Michigan

## Abstract

**Importance:**

Students who ride older school buses are often exposed to high levels of exhaust during their commutes, which may adversely affect health and school attendance. As a result, the US Environmental Protection Agency (EPA) has awarded millions of dollars to school districts to replace older, highly polluting school buses with newer, cleaner buses.

**Objective:**

To leverage the EPA’s randomized allocation of funding under the 2012-2016 School Bus Rebate Programs to estimate the association between replacing old, highly polluting buses and changes in district-average standardized test scores.

**Design, Setting, and Participants:**

This study examined changes in reading and language arts (RLA) and math test scores among US school district applicants to the EPA’s 2012-2016 national School Bus Rebate Programs 1 year before and after each lottery by selection status. Data analysis was conducted from January 15 to July 30, 2023.

**Exposure:**

Selection to receive EPA funding to replace older school buses with newer, cleaner alternatives.

**Main Outcomes and Measures:**

School district changes in RLA and math test scores among students in grades 3 through 8 before and after the EPA funding lotteries by selection status were measured using an intention-to-treat approach.

**Results:**

This study included 1941 school district applicants to the 2012-2106 EPA School Bus Rebate Programs. These districts had a mean (SD) of 14.6 (33.7) schools per district, 8755 (23 776) students per district, and 41.3% (20.2%) of students with free lunch eligibility. Among the applicants, 209 districts (11%) were selected for the clean bus funding. District-average student test scores did not improve among selected districts overall. In secondary analyses, however, districts replacing the oldest, highest polluting buses (ie, pre-1990) experienced significantly greater improvements in district-average test scores in the year after the lottery for RLA and math (SD improvement in test scores, 0.062 [95% CI, 0.050-0.074] and 0.025 [95% CI, 0.011-0.039], respectively) compared with districts without replacements.

**Conclusions and Relevance:**

In this study, the EPA funding was not associated with student test scores overall, but in secondary analyses, the replacement of the oldest school buses was associated with improved educational performance. These findings support prioritizing clean bus replacement of the oldest buses as an actionable way for improving students’ educational performance.

## Key Points

**Question:**

Is the replacement of older, more polluting school buses with newer, cleaner alternatives associated with better student educational performance?

**Findings:**

In this study of 1941 school districts that were randomly allocated funding under the 2012-2016 School Bus Rebate Programs, district-average student test scores in reading and language arts and math did not improve among selected districts overall. In secondary analyses, however, districts that were awarded funding to replace the oldest and highest-polluting buses experienced significantly greater improvements in district-average student test scores compared with those not selected for replacement funding.

**Meaning:**

These findings suggest that funding the replacement of older, more polluting school buses may be an actionable way for school districts to improve educational performance.

## Teaser

This cohort study examines the association between US Environmental Protection Agency rebate–funded replacement of older, more polluting school buses and student test scores.

## Introduction

Students riding older school buses are often exposed to high levels of diesel exhaust as a result of emissions infiltrating the bus through leaky cabins or entering the bus directly through open windows or doors.^1,2,3,4,5^ Measurements inside school buses suggest that pollutant levels can be up to 10 times higher than in outdoor air.^1,2,3^ Consequently, a student’s school bus commute may be a dominant contributor to their daily air pollution exposure.^6^ Exposure to traffic-related pollutants has been reported to be associated with adverse health outcomes^7^ and increased school absenteeism,^8,9^ which may lower educational performance.^10,11,12,13^

These findings suggest that older school buses should be replaced with newer, cleaner vehicles. Clean air technologies, such as diesel particulate matter filters, diesel oxidation catalysts, and closed crankcase ventilation systems, can lower particulate matter bus emissions by 60% to 90%^14^ and reduce particle concentrations in bus cabins by 50% to 60%.^15,16,17^ However, buses with these technologies can be costly,^18,19^ so the average school bus in the US is driven for 16 years before being retired.^20^ Thus, millions of students continue to ride school buses to school each day^21^ that are highly polluting.^22,23^

To help school districts overcome the financial obstacles of upgrading older school buses with newer, cleaner vehicles, the US Environmental Protection Agency (EPA) began awarding funding to school districts in 2012 to replace or retrofit old, highly polluting school buses. These funds were allocated randomly to applicants under the National Clean Diesel Rebate Program, which was authorized by the Diesel Emissions Reduction Act of 2010.^24^ Since then, this ongoing program has awarded an average of nearly $7 million per year for school bus upgrades.^24,25,26,27,28,29,30,31^

While earlier work suggested improvements in health, attendance, and educational performance from local school bus retrofit programs,^17,32,33^ these findings are from observational designs and only 2 states. Our earlier work documented attendance benefits of the EPA’s School Bus Rebate Program,^34^ using a randomized controlled trial design, especially when replacing the oldest buses, but data were previously unavailable to assess the educational performance that may have benefited from the program. Here, we extend our earlier work to assess EPA school bus upgrade funding and students’ educational performance.

## Methods

In this study, we compared the change in school district–level average standardized test scores of students before vs after funding for the 2012 through 2016 lotteries between districts that were and were not awarded funding. (There was no funding in 2013; however, for conciseness, we refer to 2012-2016 as our analysis years.) The University of Michigan institutional review board determined that this work was not regulated because it did not involve human participants. This study followed the Strengthening the Reporting of Observational Studies in Epidemiology (STROBE) reporting guideline.

Since test scores were only available for school districts and not school bus riders, the overall effect size associated with being selected for funding may be diluted, even though school bus emissions may affect nonriders by idling near to where students play, study, and/or wait for other transportation.^35,36,37^ Therefore, in an a priori–determined secondary analyses, we conducted our modeling by quartiles of estimated ridership on applicant buses and by model years of replaced buses since emissions standards for vehicles have strengthened over time.^38^

### Program Evaluated

The EPA’s School Bus Rebate Program, which began in 2012, provides funding to replace older diesel-powered school buses with new diesel, alternate fuel, hybrid, or electric school buses.^24,25,26,27,28,29,30,31^ The program was expanded in 2015 to include funding for retrofits of school buses with diesel oxidative catalysts and closed crankcase ventilation systems.^26,27,28^ In 2016, further funding was added to purchase EPA-verified fuel operated heaters to reduce idling for heat (Table 1; eTable 1 in Supplement 1).^27^

**Table 1.** Summary of the EPA School Bus Rebate Program by Year^a^

Lottery year	Diesel bus engines to be replaced	Replacement bus engines	No. of applications allowed	No. of buses eligible per application	Rebate amount per bus, $	No. of applicants selected for clean bus funding	No. of applicants not selected for clean bus funding	Total funding awarded, $
2012	1994-2003	2012 or later	1	5	20 000-30 000	36	973	1 880 000
2014	≤2006	2014 or later	1	5	15 000-25 000	73	474	3 940 000
2015	≤2006	2015 or later	1 (if fleet ≤100 buses) or 2 (if fleet >100 buses)	10	15 000-25 000	86	451	6 040 000
2016	≤2006	2016 or later	1 (if fleet ≤100 buses) or 2 (if fleet >100 buses)	10	15 000-25 000	92	422	7 240 000
Total	NA	NA	NA	NA	NA	287	2320	19 100 000

Abbreviations: EPA, US Environmental Protection Agency; NA, not applicable.

^a^ A version of this table was previously published in Pedde et al.^34^

School districts and private bus transportation companies servicing school districts were eligible to apply for funding to replace up to 5 or 10 buses depending on the program year. In the later program years, applicants could submit up to 2 applications depending on their fleet size. Each year had requirements regarding the model years eligible to be replaced and the type and model year of the replacement engines (Table 1; eTable 1 in Supplement 1). There were no restrictions on the number of years applicants could enter the funding lottery.

Applications were due to the EPA at the end of the calendar year for each rebate lottery. The EPA then used a random number generator to select applicants for funding until all available funds were exhausted. Beginning in 2014, some EPA regional offices used the original randomized rank of applicants that did not receive funding to award additional school bus replacement funding (eTable 2 in Supplement 1).

Applicants were notified of their funding selection status at the end of the school year, and selected applicants used their funds in the summer such that the upgraded buses were used in the fall. For example, applicants selected in the 2012 funding lottery replaced or retrofitted their buses in the summer of 2013. For 2012 applicants, we therefore refer to the 2012-2013 school year as the before year and the 2013-2014 school year as the after year. Selected applicants were responsible for any funds needed to purchase or retrofit a bus beyond what the EPA award covered. Selected applicants had to submit proof of purchase for the new buses and evidence that the old buses were scrapped.

### EPA Funding Applicant Data

We obtained data on all 2012-2016 applicants from the EPA through a Freedom of Information Act request. The collected data included lottery selection status, school district served, number of buses requested to be replaced, and amount of funding requested. For districts awarded funding, we also received information on the number of buses or engines replaced or retrofitted and the engine model year of the replaced (ie, baseline) buses, although this information was often averaged across the district’s replaced buses (eFigure 1 in Supplement 1).

### School District Educational Test Score Data

We obtained school district test score data for math and reading and language arts (RLA) for children in grades 3 through 8 from the Stanford Education Data Archive (SEDA).^39^ The SEDA harmonizes measurements from state-level testing data, which are collected under federal mandate through the Elementary and Secondary Education Act and the former No Child Left Behind Act.^40^ Under these acts, states can use a test of their choice that measures student achievement relative to the state’s own proficiency standards, which are then submitted to the US Department of Education by district, grade, and subject. To account for differences in testing between states and across time, experts at the Stanford Center for Educational Policy Analysis generated normalized educational performance metrics from these data that allow for comparisons across states and place.^40^ The SEDA also restricts its data to reliable scores, with exclusions for low participation or inconsistent assessments across students within the state, subject, and grade year.^40^ In this analysis, we averaged SEDA data across grades by district and year.

### School District Information

We gathered school districts’ demographic data from the Department of Education Local Education Agency (School District) Universe Survey Data. These data were used to compare the randomly selected and unselected districts at baseline and include the number of students (total and by grade and race and ethnicity) and schools, as well as the urbanicity (ie, city, suburb, town, rural) of each district. Race and ethnicity were self-reported by students using Department of Education–defined categories (American Indian or Alaska Native, Asian, Black or African American, Hispanic, Native Hawaiian or Pacific Islander, White, and multiracial). We obtained data on the geographic size of each district from the National Center for Education Statistics. To reflect district socioeconomic status, we aggregated school-level free or reduced-price lunch eligibility data from the annual Public Elementary/Secondary School Universe Survey Data to the district level.

### Data Exclusions

Across all 4 lottery years, there were 2607 applicants to the EPA School Bus Rebate Program (Table 1). We restricted analyses to applicants that served individual school districts because they can be linked to educational test score data; therefore, we excluded private bus transportation companies (n = 26) and school district consortium applicants (n = 13) that represented multiple school districts. We also excluded applicants that represented private (n = 5), nontraditional (eg, special education, technology centers) (n = 19), and tribal (n = 6) schools where educational testing data were not consistently available. We excluded applicants outside the continental US (n = 16) since SEDA reports test scores at the state level for Hawaii and the territory level for Puerto Rico and there were no applicants from Alaska or other US territories. After accounting for these data exclusions and test score data availability, we evaluated associations using data from 74% of the applicants (eFigure 2 in Supplement 1).

### Statistical Analysis

The EPA’s randomization was used to help ensure that the districts that received and did not receive funding were comparable with respect to measured and unmeasured characteristics. To check this balance, we compared the selected and unselected districts’ baseline characteristics using the standardized mean difference for all continuous variables. Then, we compared the continuous mean test scores (either RLA or math) for each school district *i* in the year after the year *t* lottery (*Education_it + 1_*) by selection status as described in the Model later in this paragraph. Using an intention-to-treat approach for all districts with complete data, we compared school districts *i* that were selected to receive funding (*Selected_it_* = 1) in lottery year *t* with those that were not (*Selected_it_* = 0), retaining the benefits of randomization. In addition, we adjusted for the mean test score for school district *i* in the school year of lottery *t* prior to when the new buses were in use (*Education_it_*) to account for any time-invariant differences that occurred by chance between the selected and unselected districts. This adjustment focuses on within-area differences between the pre- and postrandomization levels.^41,42^ We also included a *MultiEntrant_it_* indicator to account for some applicants submitting 2 applications within a lottery year and a *Region_i_* indicator since some EPA regions provided additional clean bus funding. To maximize power, we combined data from all lottery years but included a fixed effect for lottery year (*Time_it_*). Since school districts can enter the lottery in multiple years, we estimated associations and 95% CIs using general estimating equations with robust SEs clustered at the state level to account for any potential correlation in the data. The parameter *β_1_* in the Model is the model outcome of interest:

*Education_it_*_ + _*_1_* = *β_0_*_ _+_ _*β_1_Selected_it_* + *β_2_Education_it_* + *β_3_MultiEntrant_it_* + *βRegion_i_* + *βTime_it_*_ + _*ϵ_it_*.

Our primary analysis used district-average test scores based on data availability. This aggregation may dilute the associations between having a new bus and student achievement. While we did not have national data on school bus riders, we note that nonriders may be exposed to school bus emissions when buses idle near where students play, study, or wait for other transportation.^35,36,37^ To address this issue, we used an interaction term to evaluate effect size modification of our main association by the fraction of children likely riding the buses requested for replacement. Without data on school bus ridership rates at the district level, we estimated this fraction by multiplying the number of buses requested for replacement by 72 (the capacity for a standard school bus) and dividing by the total student enrollment for a district at baseline.

We further evaluated heterogeneity of the educational performance results by age of the replaced buses since emissions standards became more strict over time.^38^ To do so, we replaced the *Selected_it_* indicator in the Model with indicators for selected applicants who replaced pre-1990, 1990-1999, or 2000 and newer model year buses. Finally, we tested the sensitivity of our primary and model year results by alternatively modeling the difference in standardized test scores before and after the lottery as our outcome rather than controlling for the prior year’s score and with adjustment for free and reduced-price lunch eligibility.

We used SAS, version 9.4 (SAS Institute Inc) for all data processing and analyses, which were conducted from January 15 to July 30, 2023. We estimated more than 80% power to detect a difference in educational performance in RLA of 0.15 SDs. A 2-sided *P* < .05 was considered statistically significant.

## Results

We investigated 1941 school districts that applied to the 2012-2106 EPA School Bus Rebate Programs, which had a mean (SD) of 14.6 (33.7) schools per district, 8755 (23 776) students per district, and 41.3% (20.2%) of students with free lunch eligibility. Among the school districts that met inclusion criteria and had educational attainment data (74% of all applicants), 209 districts (11%) were selected for the EPA clean school bus funding (Table 2); however, 25 (12%) of these ultimately did not purchase a clean bus due to difficulty acquiring matching funds. All selected districts used the awarded funding for school bus replacements (eTable 3 in Supplement 1), with most replacing buses that were model year 2000 or newer and fewer from the 1990s and before (eFigure 1 in Supplement 1). Selected and unselected districts were similar in terms of the number of schools, geographic size, demographics, and free or reduced-price lunch eligibility (Table 2). Districts not selected for funding, however, had fewer schools (mean [SD], 14.5 [33.7] vs 15.6 [33.7]) and fewer students (mean [SD], 8634 [23 646] vs 9749 [24 861]) and requested replacement funding for fewer buses (mean [SD], 3.5 [2.6] vs 3.8 [3.0]). The RLA test scores were similar between selected and nonselected districts before buses were purchased (mean [SD], 0.067 [0.293] vs 0.065 [0.308] SDs), though districts not selected for funding had higher math test scores before the awards were made (mean [SD], 0.060 [0.335] SDs vs 0.040 [0.352] SDs).

**Table 2.** Characteristics of School District Applicants at Baseline by Lottery Status^a^^,^^b^

Characteristic	Mean (SD)		SMD^c^
	Applicants not selected for clean bus funding (n = 1732)	Applicants selected for clean bus funding (n = 209)	
No. of schools in district	14.5 (33.7)	15.6 (33.7)	0.03
No. of students in district	8634 (23 646)	9749 (24 861)	0.05
District students’ race and ethnicity, %			
Hispanic	10.7 (15.3)	12.4 (16.5)	0.10
Non-Hispanic American Indian or Alaska Native	1.4 (5.9)	1.3 (4.7)	0.01
Non-Hispanic Asian	2.1 (4.2)	2.6 (7.3)	0.09
Non-Hispanic Black or African American	9.6 (16.8)	8.8 (16.4)	0.05
Non-Hispanic Native Hawaiian or Pacific Islander	0.2 (0.4)	0.2 (0.3)	0.05
Non-Hispanic White	73.5 (25.1)	72.1 (25.8)	0.05
Non-Hispanic multiracial	2.7 (3.1)	2.7 (2.6)	0.01
District students eligible for free lunch, %	41.3 (20.0)	41.7 (21.1)	0.02
District students eligible for reduced-price lunch, %	8.0 (4.3)	8.1 (6.1)	0.01
No. of buses requested for replacement or retrofit	3.5 (2.6)	3.8 (3.0)	0.11
Funding requested for replacement or retrofit, $	77 520 (57 256)	79 043 (61 734)	0.03
District average standardized test scores, SD			
RLA before^d^	0.067 (0.293)	0.065 (0.308)	0.01
Math before^d^	0.060 (0.335)	0.040 (0.352)	0.06
RLA after^e^	0.059 (0.292)	0.056 (0.313)	0.01
Math after^e^	0.042 (0.337)	0.032 (0.368)	0.03
RLA score change^f^	−0.009 (0.095)	−0.009 (0.085)	0.01
Math score change^f^	−0.018 (0.099)	−0.009 (0.089)	0.10
District land area, square miles	263 (436)	257 (395)	0.01
District urbanicity, No. (%)			
Rural	751 (43.4)	92 (44.0)	NA
Town	407 (23.5)	39 (18.7)	NA
Suburb	412 (23.8)	57 (27.3)	NA
City	162 (9.4)	21 (10.1)	NA

Abbreviations: NA, not applicable; RLA, reading and language arts; SMD, standardized mean difference.

^a^ For applicants ultimately included in the analytic sample.

^b^ Baseline is the school year before the new buses were (or would have been in the case of unselected applicants) purchased and therefore differs by which year(s) an applicant entered the lottery.

^c^ An SMD greater than 0.10 is generally considered an indication of lack of balance. Here, we knew that districts with larger fleets could submit more applications, resulting in a greater likelihood of funding, so we included a fixed effect in our models to account for this effect.

^d^ The test score is at baseline, or in the school year before the lottery.

^e^ The test score is in the after year, or the school year after the lottery.

^f^ The test score is the change from the after year minus the before year.

Districts randomly selected for School Bus Rebate Program funding had comparable changes in educational test scores for RLA and math in the year after the lottery to districts not selected for funding (mean SD change in scores, 0.005 [95% CI, −0.007 to 0.018] higher for RLA and −0.001 [95% CI, −0.011 to 0.010] lower for math) (Figure). For districts replacing the oldest buses (pre-1990 models), however, we observed significantly larger SD improvements in mean RLA test scores of 0.062 (95% CI, 0.050-0.074) and math test scores of 0.025 (95% CI, 0.011-0.039) compared with districts without replacements. These results were robust to using the change in standardized test score as the dependent variable and adjusting for socioeconomic covariates, such as free and reduced-price lunch eligibility (Table 3).

**Figure.**
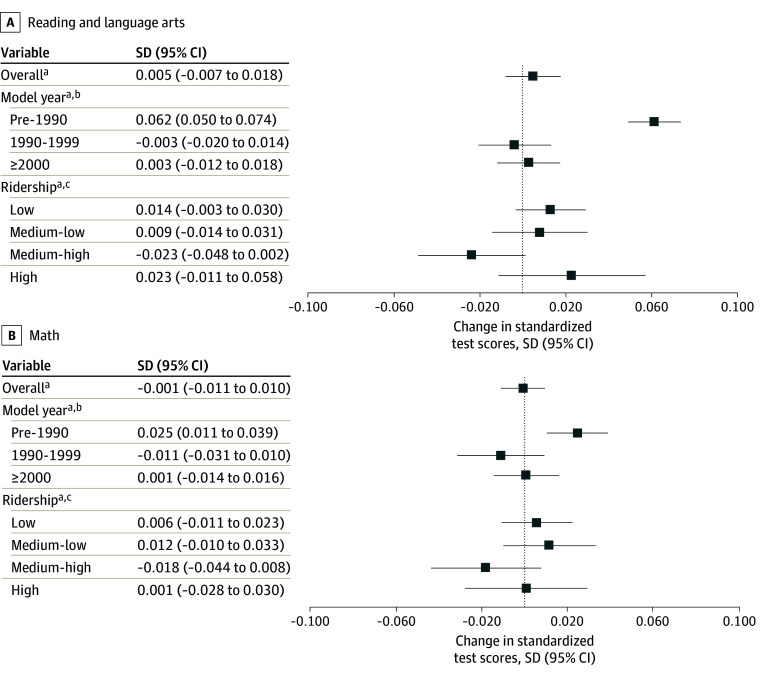
Changes in Reading and Language Arts and Math Standardized Test Scores Between Districts Awarded and Not Awarded Funds to Replace Buses Overall and by Model Year and Ridership of the Replaced Buses Higher values reflect better performance. ^a^The dependent variable is the standardized test score in the year after the lottery. The model is adjusted for standardized test score in the year before the lottery, as well as US Environmental Protection Agency region, lottery year, and an indicator for having more than 1 application in a given lottery year. ^b^The independent variables of interest are 3 indicator variables for selected applicants who replaced pre-1990, 1990-1999, or 2000 and newer model year buses. ^c^The *P* value for the interaction term by quartiles of ridership on requested buses was .14 for reading and language arts and .40 for math.

**Table 3.** Sensitivity Analyses of the Overall and Model Year–Specific Outcomes of Clean Buses on Standardized Test Scores

Analysis	Parameter estimate (95% CI)	
	RLA	Math
**Primary model** ^a^		
Overall outcome of replacement	0.005 (−0.007 to 0.018)	−0.001 (−0.011 to 0.010)
Model year^b^		
Pre-1990	0.062 (0.050 to 0.074)	0.025 (0.011 to 0.039)
1990-1999	−0.003 (−0.020 to 0.014)	−0.011 (−0.031 to 0.010)
≥2000	0.003 (−0.012 to 0.018)	0.001 (−0.014 to 0.016)
**Change in standardized test score** ^c^		
Overall outcome of replacement	0.006 (−0.008 to 0.020)	−0.0003 (−0.011 to 0.010)
Model year^b^		
Pre-1990	0.068 (0.054 to 0.081)	0.028 (0.014 to 0.042)
1990-1999	−0.004 (−0.020 to 0.013)	−0.012 (−0.031 to 0.008)
≥2000	0.003 (−0.013 to 0.019)	0.001 (−0.014 to 0.016)
**Adjustment for socioeconomic status** ^d^		
Overall outcome of replacement	0.005 (−0.008 to 0.019)	0.001 (−0.010 to 0.012)
Model year^b^		
Pre-1990	0.066 (0.053 to 0.079)	0.042 (0.027 to 0.057)
1990-1999	−0.002 (−0.019 to 0.016)	−0.005 (−0.024 to 0.015)
≥2000	0.003 (−0.012 to 0.019)	−0.001 (−0.018 to 0.016)

Abbreviation: RLA, reading and language arts.

^a^ The dependent variable is the standardized test score in the year after the lottery. The model is adjusted for standardized test score in the year before the lottery, as well as US Environmental Protection Agency (EPA) region, lottery year, and an indicator for having more than 1 application in a given lottery year.

^b^ The independent variables of interest are 3 indicator variables for selected applicants who replaced pre-1990, 1990-1999, or 2000 and newer model year buses.

^c^ The dependent variable is the difference in standardized test scores in the years before and after the lottery. The model is adjusted for EPA region, lottery year, and an indicator for having more than 1 application in a given lottery year.

^d^ The dependent variable is the standardized test score in the year after the lottery. The model is adjusted for free lunch eligibility and reduced-price lunch eligibility, as well as for standardized test score in the year before the lottery, EPA region, lottery year, and an indicator for having more than 1 application in a given lottery year.

## Discussion

In this assessment of educational performance and the EPA’s School Bus Rebate Program, standardized test scores did not improve in school districts randomly selected for funding overall. In secondary analyses, however, school districts awarded funding to upgrade the oldest, most polluting school buses had greater improvements in RLA and math standardized test scores than districts not awarded funding to replace their buses. Funding was not associated with greater improvements in test scores among districts who replaced buses from 1990 or later. This suggests that there could be educational benefit to school children from decommissioning pre-1990 model year school buses.

An important feature of this work is the randomized allocation of the EPA’s clean school bus funding. This design is notably different from previous studies^32,33^ that relied on comparisons between school districts that self-selected to replace buses or not and the potential for bias and uncontrolled confounders in these studies. Randomization should remove any systematic differences by measured or unmeasured characteristics between districts and ensure that those with and without funding are comparable. Our study is the first in our knowledge to evaluate educational performance and the EPA’s national School Bus Rebate Program. Given that our group previously found that cleaner buses were associated with better respiratory health in children^17^ and greater attendance rates,^34^ these findings are consistent with considerable evidence that school attendance is associated with student achievement.^43,44,45,46,47,48^ There is also evidence that air pollution is directly associated with cognitive performance in children,^49,50,51,52^ providing another possible mechanism with which to interpret our important results.

Our finding that the greatest educational improvements were observed in districts replacing the oldest buses is consistent with our earlier work that also found the greatest improvements in attendance among districts that replaced the oldest buses.^34^ These findings are plausible since emissions standards for school buses have strengthened over time, with the largest changes in 1991 followed by weaker changes in 1998, 2004, and 2007.^38^ While strengthening of emissions standards over time means that fewer old buses may be in circulation in the future, we estimate that there are nearly 5000 pre-1990 buses in the US fleet^22,23,53^ and, thus, nearly 250 000 students on these buses.^54^ At a cost of up to $300 000 per bus,^19^ an investment of $1.5 billion could replace all older buses, which equals approximately one-third of the budget already allocated to EPA’s new Clean School Bus Program over the next 5 years to purchase zero- and low-emission vehicles.^55^ Importantly, districts with the oldest buses might also have the least resources to upgrade buses absent the EPA funds.

Overall, our findings are consistent with another study by Austin et al^32^ that estimated associations between school bus retrofits and educational performance. The authors found that school bus retrofits in Georgia were associated with significant improvements on standardized testing. Specifically, they found that districts retrofitting 19% of their fleet (the mean in their sample) had mean increases in English language arts and math scores of 0.017 SDs (95% CI, 0.006-0.028 SDs) and 0.009 SDs (95% CI, −0.002 to 0.020 SDs), respectively. These findings are smaller than ours for the replacement of the oldest buses (mean SD increase, 0.062 [95% CI, 0.050-0.074] and 0.025 [95% CI, 0.011-0.039] for RLA and math, respectively) but stronger than our overall findings (mean, 0.005 SDs [95% CI, −0.007 to 0.018 SDs] higher average RLA test scores and −0.001 SDs [95% CI, −0.011 to 0.010 SDs] lower math test scores), which may suggest an older fleet in this area or a larger fraction of district fleets being affected. Our findings are also supported by the broader literature that has reported associations of higher traffic-related air pollution in outdoor air with poorer academic performance,^10,11,12^ though the quality of the evidence has been assessed as generally low.^13^

While EPA clean bus funding has increased over time (Table 1), we adjusted for time trends in our models so that this should not introduce bias. Relatedly, additional funding from some EPA regional offices was a possible concern, but the use of the EPA’s original randomized ranking to distribute the additional funds to applicants not selected by the national program may mitigate this concern, as did our adjustment for EPA region. Even with these increases, relatively few total applicants were ultimately awarded funding, which reduced the power of our analysis. While we had intended to include the 2017 and 2018 lotteries in this analysis, the educational performance data were not yet available for the after year of the 2017 lottery, and academic testing during the after year of the 2018 lottery was disrupted by the COVID-19 pandemic.

### Limitations

There are several limitations of this work. We did not have information on which schools within a district the buses serviced, and the educational achievement data were district-wide (ie, not only among bus riders) for students in grades 3 through 8. These factors may have influenced our results if, for example, all buses were used to transport high school students or there was a substantial number of children not riding the replaced buses. These limitations may explain our null findings in the overall sample. As such, we found no evidence of a dose-response association across quartiles of estimated bus ridership levels (Figure), suggesting that the dilution hypothesis due to ridership may be unlikely. A related limitation is that since the educational data are only derived from students in grades 3 through 8, our results may not extrapolate to students outside of this age range.

Another limitation is our use of an intention-to-treat analysis. This approach assumes that any district selected for funding replaced their buses and those not selected for funding did not replace their buses. However, we know that there were 25 districts selected for funding that ultimately did not purchase a clean bus. We have no information on whether districts not selected for funding replaced their buses, but the possibility exists. While the intention-to-treat analysis is the standard approach for randomized controlled studies since it retains the benefits of randomization, nonadherence may bias our results toward the null. Thus, the true association between being selected in the EPA’s clean school bus funding lottery and educational performance may be higher than what is reported here.^56^ We also note that since applicants that applied for the EPA funding needed to fund the remaining costs of bus replacements or retrofits beyond the EPA award, our findings may not be generalizable to all districts.

In addition, our inclusion criteria were applied after the randomization. It was not expected that this procedure would distort the randomization, however, since we found no evidence that being excluded was associated with being selected in the lottery. This finding greatly mitigates concerns of selection bias due to the exclusion of applicants post randomization.

## Conclusions

Although we did not find evidence that the EPA’s School Bus Rebate Program was associated with improved district-level student academic performance, districts replacing the oldest buses had greater improvements in educational standardized test scores than districts not awarded funding. Given the importance of education to both individuals and society, we conclude that the replacement of older school buses may be an actionable way for school districts to improve educational performance.

## Notes

Supplement 1. **eTable 1.** EPA School Bus Rebate Program Details by Year **eTable 2.** Number of School District Lottery Applicants by EPA Region and Number of Applicants (%) Receiving EPA School Bus Rebate Program Funding by Source of Funding by Year **eTable 3.** Types of School Bus Upgrades Purchased by EPA School Bus Rebate Program Awardees, 2012-2016 **eFigure 1.** Histogram of Applicant Average Model Year of Replaced Buses for 2012-2016 Applicants Selected for EPA Funding **eFigure 2.** Flow Chart Documenting Exclusion Criteria and the Number of Applicants Included in the Study Analyses

Supplement 2.
Data Sharing Statement
