# Supplementary material for: School Bus Rebate Program and Student Educational Performance Test Scores
Source: JAMA Netw Open. 2024 Mar 20;7(3):e243121. doi: 10.1001/jamanetworkopen.2024.3121 (PMC10955349; doi:10.1001/jamanetworkopen.2024.3121)
Supplement: Supplement 1. — eTable 1. EPA School Bus Rebate Program Details by Year eTable 2. Number of School District Lottery Applicants by EPA Region and Number of Applicants (%) Receiving EPA School Bus Rebate Program Funding by Source of Funding by Year eTable 3. Types of School Bus Upgrades Purchased by EPA School Bus Rebate Program Awardees, 2012-2016 eFigure 1. Histogram of Applicant Average Model Year of Replaced Buses for 2012-2016 Applicants Selected for EPA Funding eFigure 2. Flow Chart Documenting Exclusion Criteria and the Number of Applicants Included in the Study Analyses [file jamanetwopen-e243121-s001.pdf]

Supplemental Online Content

Pedde M, Szpiro A, Hirth RA, Adar SD. School bus rebate program and student educational performance test scores. *JAMA Netw Open*. 2024;7(3):e243121. doi:10.1001/jamanetworkopen.2024.3121

eTable 1. EPA School Bus Rebate Program Details by Year

eTable 2. Number of School District Lottery Applicants by EPA Region and Number of Applicants (%) Receiving EPA School Bus Rebate Program Funding by Source of Funding by Year

eTable 3. Types of School Bus Upgrades Purchased by EPA School Bus Rebate Program Awardees, 2012-2016

eFigure 1. Histogram of Applicant Average Model Year of Replaced Buses for 2012-2016 Applicants Selected for EPA Funding

eFigure 2. Flow Chart Documenting Exclusion Criteria and the Number of Applicants Included in the Study Analyses

This supplemental material has been provided by the authors to give readers additional information about their work.

© 2024 Pedde M et al. *JAMA Network Open.*

eTable 1. EPA School Bus Rebate Program Details by Year[a]

Lottery Year	Program Details	Funding by Vehicle Class	Retrofit Details
2012	Replacements only. New buses powered by a certified 2012 or newer model year engine, or operate solely on electricity. Eligible replacement school buses may operate on conventional diesel (ULSD), battery or hybrid drivetrains, or alternative fuels.	Class 3-5: $20K<br>Class 6-7: $25K<br>Class 8: $30K	
2014	Replacements only. New buses powered by a certified 2014 or newer model year engine, or operate solely on electricity. Eligible replacement school buses may operate on conventional diesel (ULSD), battery or hybrid drive trains, or alternative fuels.	Class 3-5: $15K<br>Class 6-7: $20K<br>Class 8: $25K	
2015	Replacements and Retrofits. New buses powered by a certified 2015 or newer model year engine, or operate solely on electricity. Eligible replacement school buses may operate on conventional diesel (ULSD), battery or hybrid drive trains, or alternative fuels.<br>Retrofits must be 1994-2006 model year engine powered by ULSD.	Class 3-5: $15K<br>Class 6-7: $20K<br>Class 8: $25K	Retrofits: up to $3K for DOC+CCV (per bus)
2016	Replacements and Retrofits. New buses powered by a certified 2016 or newer model year engine, or operate solely on electricity. Eligible replacement school buses may operate on conventional diesel (ULSD), gasoline, battery or hybrid drivetrains, or alternative fuels. Retrofits must be 1994-2006 model year engine powered by ULSD. Encouraged fuel-operated heaters for both replacements and retrofits	Class 3-5: $15K<br>Class 6-7: $20K<br>Class 8: $25K	Retrofits: up to $4K for DOC+CCV, $6K for DOC+CCV+FOH (all per bus)

Abbreviations: EPA, United States Environmental Protection Agency; ULSD, ultra-low-sulfur diesel fuel; K, thousand; DOC, diesel oxidation catalysts; CCV, closed crankcase ventilation system; FOH, fuel operated heater.
[a] A version of this table was previously published in Pedde et al. 2023.[34]

© 2024 Pedde M et al. *JAMA Network Open.*

eTable 2. Number of School District Lottery Applicants by EPA Region and Number of Applicants (%) Receiving EPA School Bus Rebate Program Funding by Source of Funding by Year[a]

	Lottery Year									
	2012		2014		2015		2016		All Years[b]	
Awarding Entity	Number of School District Applicants	Number of Funded School District Applicants[c,d]	Number of School District Applicants	Number of Funded School District Applicants[c,d]	Number of School District Applicants	Number of Funded School District Applicants[c,d]	Number of School District Applicants	Number of Funded School District Applicants[c,d]	Number of School District Applicants	Number of Funded School District Applicants[c,d]
EPA Headquarters	--	28 (100%)	--	53 (75%)	--	44 (62%)	--	58 (71%)	--	183
EPA Region 1	41	0 (0%)	31	3 (4%)	17	9 (13%)	30	3 (4%)	119	15
EPA Region 2	94	0 (0%)	48	0 (0%)	54	10 (14%)	45	11 (13%)	241	21
EPA Region 3	55	0 (0%)	8	0 (0%)	18	0 (0%)	37	1 (1%)	118	1
EPA Region 4	99	0 (0%)	60	0 (0%)	108	0 (0%)	79	6 (7%)	346	6
EPA Region 5	332	0 (0%)	170	0 (0%)	99	0 (0%)	113	0 (0%)	714	0
EPA Region 6	114	0 (0%)	26	2 (3%)	26	6 (8%)	13	1 (1%)	179	9
EPA Region 7	136	0 (0%)	111	13 (18%)	108	1 (1%)	91	2 (2%)	446	16
EPA Region 8	64	0 (0%)	34	0 (0%)	32	1 (1%)	29	0 (0%)	159	1
EPA Region 9	22	0 (0%)	31	0 (0%)	41	0 (0%)	42	0 (0%)	136	0
EPA Region 10	52	0 (0%)	28	0 (0%)	34	0 (0%)	35	0 (0%)	149	0
Total:	1,009	28	547	71	537	71	514	82	2,607	252

Abbreviations: EPA, United States Environmental Protection Agency.
[a] A version of this table was previously published in Pedde et al. 2023.[34]
[b] Lottery years 2012, 2014, 2015, and 2016.
[c] Percentages represent the within-year distribution of funding sources; column percentages may not sum to 100% due to rounding.
[d] This column only summarizes the applicants who were ultimately awarded funding (i.e., it does not include the applicants (N=35) who were selected to receive funding but ultimately did not).

© 2024 Pedde M et al. *JAMA Network Open.*

eTable 3. Types of School Bus Upgrades Purchased by EPA School Bus Rebate Program Awardees, 2012-2016[a]

Technology	Number	Percent
Vehicle Replacement - ULSD (diesel)	242	96.4%
Vehicle Replacement - LPG/Propane	6	2.4%
Vehicle Replacement - CNG	3	1.2%
**Total:**	**251**	**100%**

Abbreviations: EPA, United States Environmental Protection Agency; ULSD, ultra-low-sulfur diesel fuel; LPG, liquefied petroleum gas; CNG, compressed natural gas.
[a] Information on the type of school bus upgrade purchased was only available for 251 of the total 287 selected applicants of the 2012-2016 lotteries.

© 2024 Pedde M et al. *JAMA Network Open.*

eFigure 1. Histogram of Applicant Average Model Year of Replaced Buses for 2012-2016 Applicants Selected for EPA Funding

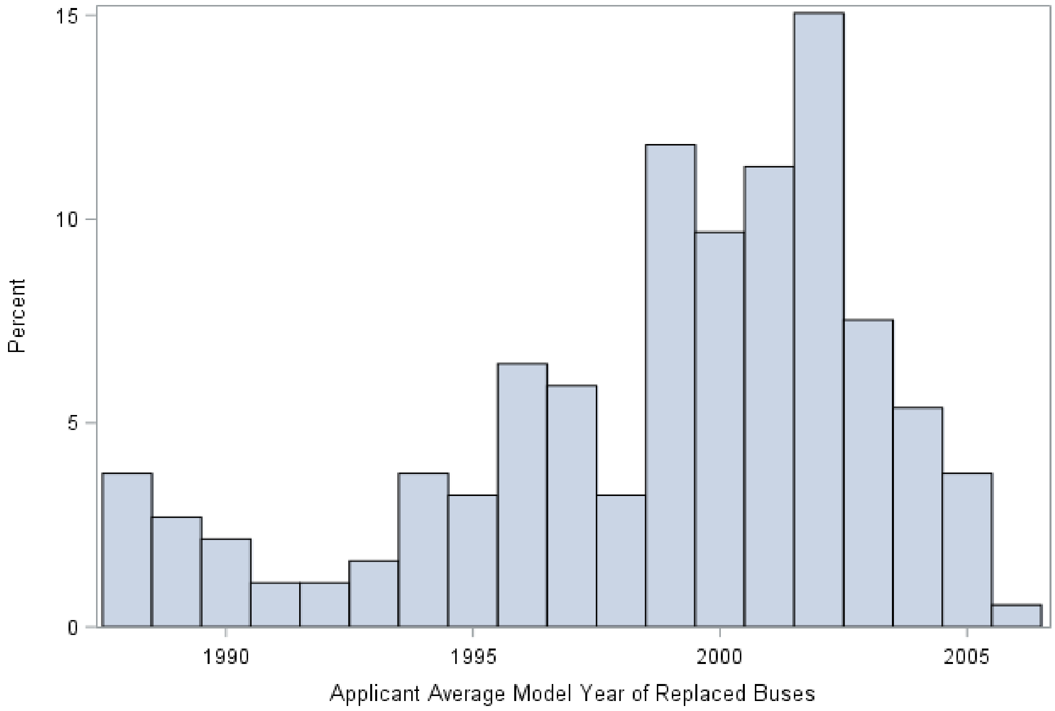

© 2024 Pedde M et al. *JAMA Network Open.*

eFigure 2. Flow Chart Documenting Exclusion Criteria and the Number of Applicants Included in the Study Analyses

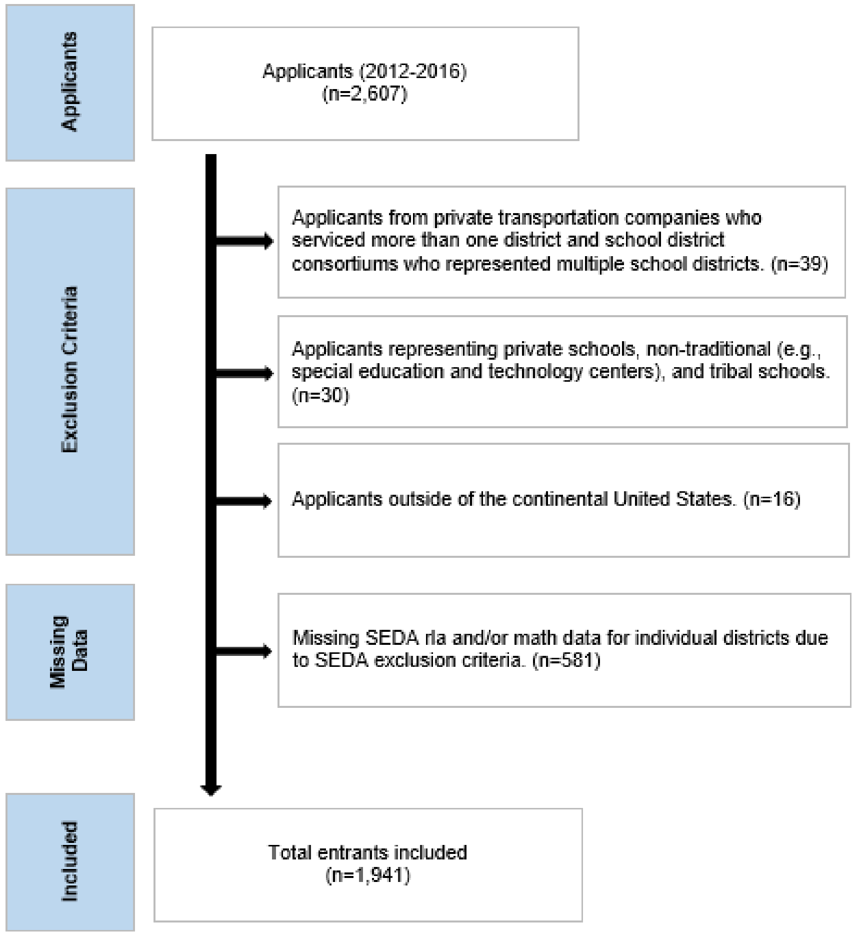

Abbreviations: SEDA, Stanford Education Data Archive.

© 2024 Pedde M et al. *JAMA Network Open.*
