# Supplementary material for: School Bus Rebate Program and Student Educational Performance Test Scores
Source: JAMA Netw Open. 2024 Mar 20;7(3):e243121. doi: 10.1001/jamanetworkopen.2024.3121 (PMC10955349; doi:10.1001/jamanetworkopen.2024.3121)
Supplement: Supplement 2. — Data Sharing Statement [file jamanetwopen-e243121-s002.pdf]

Data Sharing Statement

Pedde. School Bus Rebate Program and Student Educational Performance Test Scores. *JAMA Netw Open*. Published March 20, 2024. doi:10.1001/jamanetworkopen.2024.3121

Data
Data available: Yes
Data types: Data (not involving human participants), Data dictionary
How to access data: https://deepblue.lib.umich.edu
When available: With publication

Supporting Documents
Document types: Statistical/analytic code
How to access documents: https://deepblue.lib.umich.edu
When available: With publication

Additional Information
Who can access the data: anyone
Types of analyses: for any purpose
Mechanisms of data availability: with investigator support
Any additional restrictions: none
